# Development and Validation of a Machine-Learning Model for Prediction of Extubation Failure in Intensive Care Units

**DOI:** 10.3389/fmed.2021.676343

**Published:** 2021-05-17

**Authors:** Qin-Yu Zhao, Huan Wang, Jing-Chao Luo, Ming-Hao Luo, Le-Ping Liu, Shen-Ji Yu, Kai Liu, Yi-Jie Zhang, Peng Sun, Guo-Wei Tu, Zhe Luo

**Affiliations:** ^1^College of Engineering and Computer Science, Australian National University, Canberra, ACT, Australia; ^2^Department of Critical Care Medicine, Zhongshan Hospital, Fudan University, Shanghai, China; ^3^Shanghai Medical College, Fudan University, Shanghai, China; ^4^Department of Blood Transfusion, The Third Xiangya Hospital of Central South University, Changsha, China; ^5^Artificial Intelligence Institute, Shanghai Jiao Tong University, Shanghai, China; ^6^Department of Critical Care Medicine, Xiamen Branch, Zhongshan Hospital, Fudan University, Xiamen, China

**Keywords:** extubation failure, recursive feature elimination, hyperparameter optimization, categorical boosting, prospective validation

## Abstract

**Background:** Extubation failure (EF) can lead to an increased chance of ventilator-associated pneumonia, longer hospital stays, and a higher mortality rate. This study aimed to develop and validate an accurate machine-learning model to predict EF in intensive care units (ICUs).

**Methods:** Patients who underwent extubation in the Medical Information Mart for Intensive Care (MIMIC)-IV database were included. EF was defined as the need for ventilatory support (non-invasive ventilation or reintubation) or death within 48 h following extubation. A machine-learning model called Categorical Boosting (CatBoost) was developed based on 89 clinical and laboratory variables. SHapley Additive exPlanations (SHAP) values were calculated to evaluate feature importance and the recursive feature elimination (RFE) algorithm was used to select key features. Hyperparameter optimization was conducted using an automated machine-learning toolkit (Neural Network Intelligence). The final model was trained based on key features and compared with 10 other models. The model was then prospectively validated in patients enrolled in the Cardiac Surgical ICU of Zhongshan Hospital, Fudan University. In addition, a web-based tool was developed to help clinicians use our model.

**Results:** Of 16,189 patients included in the MIMIC-IV cohort, 2,756 (17.0%) had EF. Nineteen key features were selected using the RFE algorithm, including age, body mass index, stroke, heart rate, respiratory rate, mean arterial pressure, peripheral oxygen saturation, temperature, pH, central venous pressure, tidal volume, positive end-expiratory pressure, mean airway pressure, pressure support ventilation (PSV) level, mechanical ventilation (MV) durations, spontaneous breathing trial success times, urine output, crystalloid amount, and antibiotic types. After hyperparameter optimization, our model had the greatest area under the receiver operating characteristic (AUROC: 0.835) in internal validation. Significant differences in mortality, reintubation rates, and NIV rates were shown between patients with a high predicted risk and those with a low predicted risk. In the prospective validation, the superiority of our model was also observed (AUROC: 0.803). According to the SHAP values, MV duration and PSV level were the most important features for prediction.

**Conclusions:** In conclusion, this study developed and prospectively validated a CatBoost model, which better predicted EF in ICUs than other models.

## Introduction

Extubation, the process of removing an artificial airway to liberate a patient from mechanical ventilation (MV), leads to non-negligible risks due to significant respiratory and circulatory changes. Although MV is an advanced respiratory support widely used in intensive care units (ICUs) (1), prolonged ventilation is associated with poorer prognosis and should be avoided (2, 3). However, premature extubation in unprepared patients will cause extubation failure (EF), leading to a higher risk of ventilator-associated pneumonia, extended hospital stays, and higher mortality (25–50%) (4, 5). Therefore, it is significant to accurately predict the EF risk and optimize the timing of MV weaning.

Many factors have been assessed by prior studies for EF prediction, including Rapid Shallow Breathing Index (RSBI, f/Vt) (6), prolonged MV (7, 8), and cough strength (9, 10). Unfortunately, it was shown that these factors as well as physicians' judgments were not as accurate as expected (11, 12). As a result, the current weaning criteria based on these factors are still unsatisfactory. 10–29% of patients who have met these criteria still experience reintubation (1, 3).

With the rapid development of precision medicine, machine-learning approaches, respected as a deep analysis “vehicle,” have derived predictive tools in a vast range of clinical applications (13–15). Some previous studies have explored the ability of machine-learning models to accurately predict EF in recent years (11, 16, 17). Despite remarkable accuracy, these studies had a limited sample size, including only hundreds of observations. Although data resampling methods were applied, the models might overfit specific populations and therefore, lack generalization ability. Other studies developed models based on larger datasets, but they failed to validate their model on an external dataset (12, 18). Furthermore, score variables such as Acute Physiology Age Chronic Health Evaluation (APACHE)-II and Therapeutic Intervention Scoring System (TISS) are included in all these models, probably making the models inconvenient for use in clinical settings.

In this study, we aimed to develop and validate a machine-learning model with good accuracy for a general population. To this end, we explored a large-scale public database to develop a prediction model, using features selected according to their importance and clinical availability. In addition, our model was further validated in a university teaching hospital prospectively.

## Materials and Methods

### Source of Data

The model was developed and internally validated based on a sizeable critical care database called the Medical Information Mart for Intensive Care (MIMIC)-IV (19), which consists of comprehensive and high-quality data of patients admitted to ICUs at the Beth Israel Deaconess Medical Center between 2008 and 2019. One author (QZ) obtained access to the database and was responsible for data extraction. For external validation, a prospective cohort was developed in the Cardiac Surgical ICU (CSICU) of Zhongshan Hospital, Fudan University (ZS cohort). This cohort was approved by its institutional ethics committee (Approval No. B2019-075R). The study was reported according to the recommendations of the Transparent Reporting of a multivariable prediction model for Individual Prognosis Or Diagnosis (TRIPOD) statement (20).

### Selection of Participants

In the MIMIC-IV cohort, patients who underwent extubation during ICU stays were included. The exclusion criteria were as follows: (i) age <18 years, (ii) unplanned extubation, (iii) not the first extubation during the hospital stay, or (iv) no MV records before extubation. In the ZS cohort, all eligible patients that did not meet the exclusion criteria described above from December 2020 to January 2021, were prospectively enrolled. Written consent was obtained from patients' legally authorized representatives upon admission to the ICU.

### Data Collection and Outcome Definition

In the MIMIC-IV cohort, clinical and laboratory variables were extracted within 4 h before extubation (Supplementary Table 1), including patient characteristics (age, gender, and ethnicity), laboratory data (arterial blood gas, full blood count, liver function, and renal function), vital signs (respiratory rate, blood pressure, heart rate, peripheral oxygen saturation (SpO_2_), and temperature). For some variables with multiple measurements, average values were assessed. The average amount per hour of transfusion (red blood cells, platelets, and fresh frozen plasma) and fluid balance (urine output, crystalloid bolus, and colloid bolus) were calculated within 24 h before extubation, and were then normalized by patient weight. Comorbidities were also assessed based on the recorded International Classification of Diseases (ICD)-9 and ICD-10 codes (21), and the Charlson Comorbidity Index was calculated (22). In addition, data on medications such as heparin, antibiotics and vasopressors, as well as continuous renal replacement therapy (CRRT) were extracted. Finally, the 28-day mortality, reintubation, and initiation of non-invasive ventilation (NIV) after extubation were also assessed. In the ZS cohort, due to limited manpower, we did not collect all the variables; instead, key candidate variables were recorded when patients underwent extubation. Patients were followed up until discharge or death.

The primary outcome of the present study was EF, which was defined as the need for ventilatory support (NIV or reintubation) or death within 48 h following planned extubation (5, 23).

### Statistical Analysis

Baseline characteristics were compared between the successful extubation group and the EF group in the MIMIC-IV and ZS cohorts. For continuous variables, values are presented as the means (standard deviations) (if normal) or medians [interquartile ranges] (if non-normal), and comparisons were made using Student's *t*-test or the rank-sum test, as appropriate. For categorical variables, values are presented as total numbers [percentages] and the Chi-square test or Fisher's exact test were used, as appropriate, to examine differences between the two groups.

**Figure 1:**
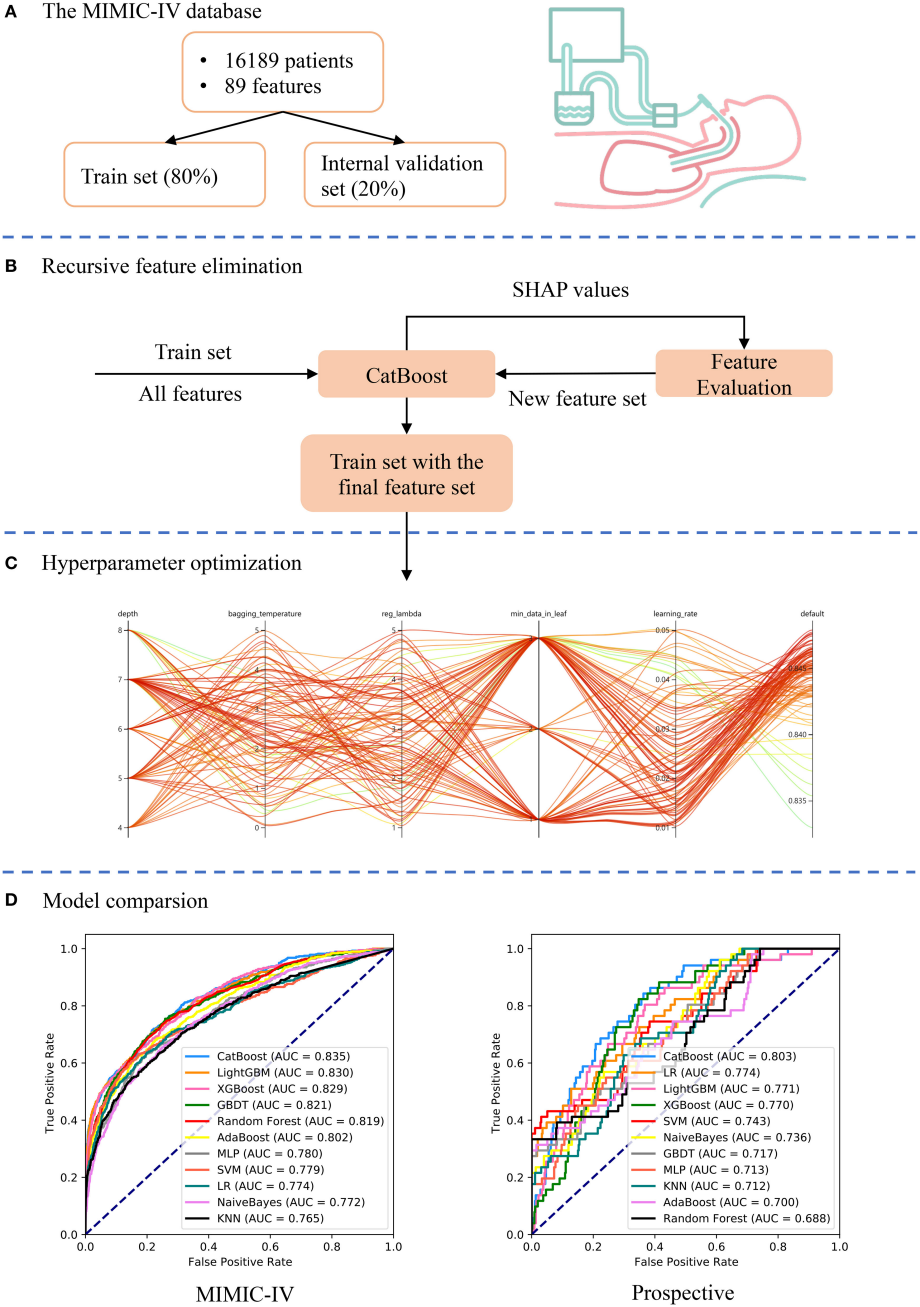
Schematic illustration of the study design. **(A)** Patients who underwent extubation in the Medical Information Mart for Intensive Care (MIMIC)-IV database were included in the study and 89 variables were extracted. The dataset was divided into train set (80%) and internal validation set (20%). **(B)** The recursive feature elimination algorithm was performed based on the train set, and key features were selected. **(C)** Hyperparameters was optimized using an automated machine learning toolkit on the train set. **(D)** The developed CatBoost model outperformed other models both in the internal validation and prospective validation sets.

An advanced machine-learning model called CatBoost was developed using the Catboost Python package (version 0.24). As shown in Figure 1, the MIMIC-IV dataset was first randomly split into the train set (80%) and internal validation set (20%). Categorical variables or missing values were not processed, as the CatBoost algorithm could handle them automatically. Second, the recursive feature elimination (RFE) algorithm based on SHapley Additive exPlanations values was performed to screen out key features, as shown in Figure 1B. Thus, a full CatBoost model was developed based on the train set with all available variables to predict EF. Second-order variables were calculated based on other variables, such as RSBI, Sequential Organ Failure Assessment (SOFA) and Simplified Acute Physiology Score (SAPS)-II, were manually excluded. The effects of remaining features on prediction scores were then measured using the functions of the SHAP Python package (version 0.32.1), which assessed the importance of each feature using a game-theoretic approach (24). The feature with the smallest effect on the prediction was eliminated in each loop, and a new CatBoost model was recursively fitted based on smaller feature sets until a significant decrease in model performance was observed (25). Finally, key features were selected that had the greatest importance and were easy to collect in clinical settings.

To further improve the model performance, hyperparameter tuning was conducted using an automated machine learning toolkit called Neural Network Intelligence (NNI) designed by Microsoft Research. We chose the Tree-structured Parzen Estimator (TPE), one of the sequential model-based optimization algorithms, as the tuning algorithm. TPE sequentially constructed models to approximate the performance of hyperparameters based on historical measurements, and then subsequently chose new hyperparameters to test based on this model (26). The hyperparameter search domain is summarized in Supplementary Table 2. One hundred trials were carried out and the parameters with the greatest area under the receiver operating characteristic (AUROC) were saved. A compact CatBoost model using the saved parameters was then trained based on the selected features, and then validated in the validation sets.

AUROCs were also calculated to compare our model and other predictive factors commonly used in the ICU, such as RSBI, SOFA, SAPS-II, and ROX (the ratio of pulse oximetry/fraction of inspired oxygen to respiratory rate). Additionally, 10 different models were derived in the train set and compared with our CatBoost model, including K-Nearest Neighbor (KNN), AdaBoost, Multi-Layer Perceptron (MLP), Support Vector Machine (SVM), Logistic Regression (LR), NaiveBayes, Gradient Boosting Decision Tree (GBDT), random forest, eXtremely Gradient Boosting (XGBoost) and LightGBM (15). Note that most of these models could not analyze data with missing values, and therefore, datasets were imputed by multiple imputation (27). In addition, categorical variables were converted to one-hot encoding and data were centered to zero and scaled before training the KNN, MLP, SVM, LR, and NaiveBayes models. These models and our CatBoost model were compared both in the internal and prospective validation sets.

All statistical analyses in the present study were performed using Python (version 3.7.6); *p* < 0.05 was considered statistically significant.

## Results

### Baseline Characteristics

**Figure 2:**
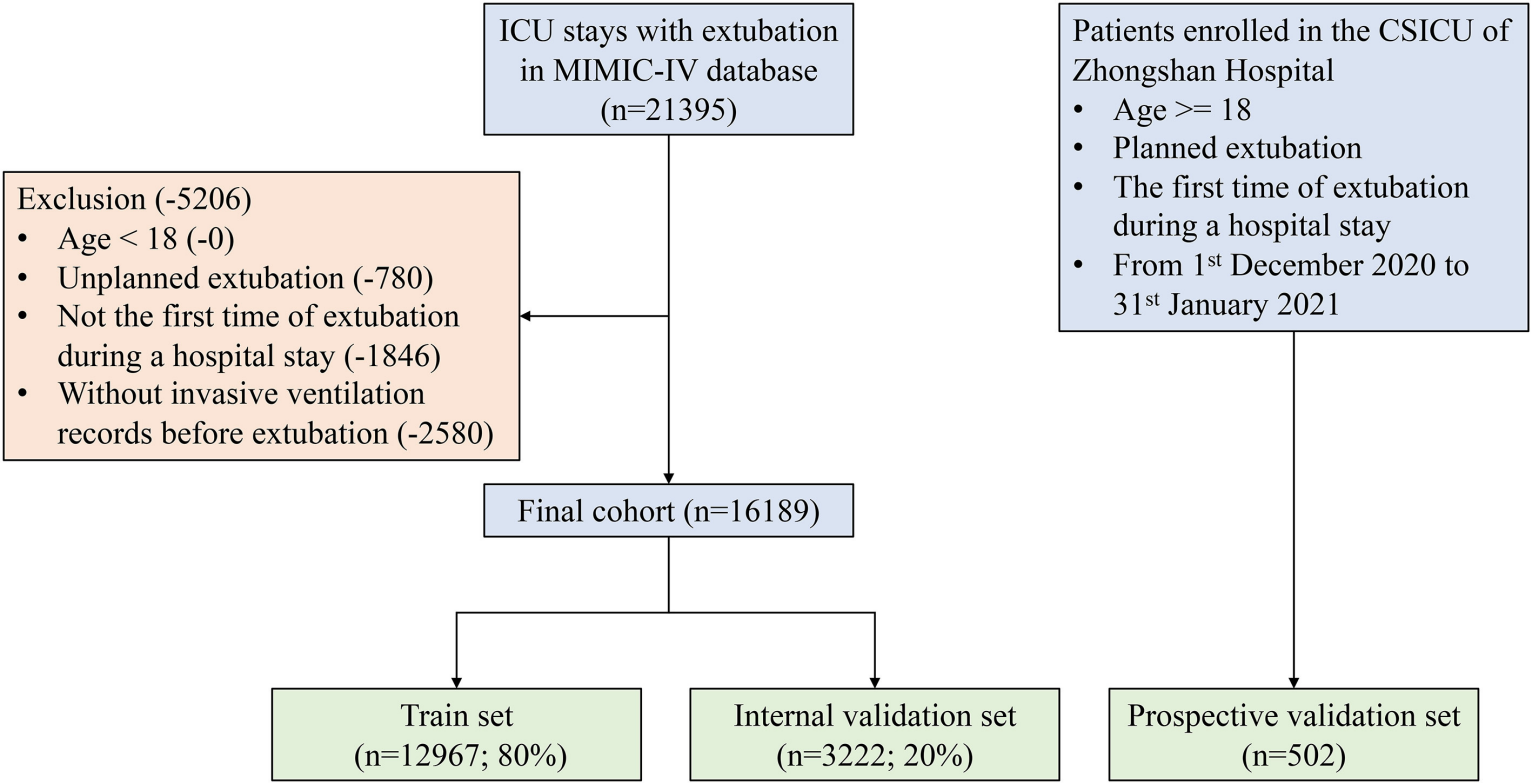
Flow chart of patient selection. MIMIC-IV, Medical Information Mart for Intensive Care-IV; ICU, intensive care unit.

As shown in Figure 2, a total of 16,189 and 502 patients who underwent extubation were ultimately included in the MIMIC-IV and ZS cohorts, respectively. The MIMIC-IV dataset was then divided into the train set (*n* = 12,967) and the internal validation set (*n* = 3,222).

A comparison of baseline characteristics between the successful extubation and EF groups in the MIMIC-IV and ZS cohorts is summarized in Table 1. In both cohorts, patients in the failure group had a higher rate of stroke, higher heart rate and respiratory rate, and mean airway pressure (*p* < 0.05). Significant prolonged MV duration and lower urine output were also observed in the failure group in both cohorts. No significant difference in pressure support ventilation (PSV) between the successful extubation and EF group was observed in the ZS cohort as a PSV level of 5 was routinely set at the beginning (28), and the level was elevated when the target tidal volume could not be reached, but not if the patients were unable to tolerate that.

**Table 1:** Baseline characteristics of the MIMIC-IV and ZS cohorts.

	**MIMIC-IV cohort**			**Zhongshan hospital cohort**		
	**Success (*****n*** **=** **13,433)**	**Failure (*****n*** **=** **2,756)**	***P*****-value**	**Success (*****n*** **=** **451)**	**Failure (*****n*** **=** **51)**	***P*****-value**
Age	64 (16)	68 (15)	<0.001	60 (13)	63 (12)	0.073
BMI	30 (7)	30 (9)	<0.001	24 (12)	26 (4)	0.135
Strokes, *n* (%)	968 (7)	543 (20)	<0.001	23 (5)	7 (14)	0.024
Heart rate (/min)	83 (15)	88 (18)	<0.001	85 (14)	95 (20)	0.002
Respiratory rate (/min)	18 (4)	20 (5)	<0.001	20 (8)	23 (6)	<0.001
MAP (mmHg)	79 (12)	76 (15)	<0.001	81 (10)	80 (15)	0.508
SpO_2_ (%), median [Q1,Q3]	99 [97,100]	98 [96,99]	<0.001	99 [98,100]	99 [97,100]	0.433
Temperature (°C)	37.0 (0.6)	37.1 (0.9)	<0.001	36.8 (0.6)	36.9 (0.7)	0.183
pH	7.39 (0.05)	7.36 (0.11)	<0.001	7.41 (0.04)	7.44 (0.03)	0.197
CVP (mmHg)	10 (4)	12 (5)	<0.001	11 (2)	12 (3)	0.125
Tidal volume (mL/kg), median [Q1,Q3]	5.8 [4.7,7.1]	5.6 [4.4,6.9]	<0.001	7.2 [6.3,8.5]	6.9 [5.5,8.2]	0.557
PEEP (cmH_2_O)	4.6 (1.7)	6.0 (3.0)	<0.001	5 (0.0)	5 (0.0)	1.000
Mean airway pressure (cmH_2_O)	7.3 (2.2)	9.3 (4.1)	<0.001	7.1 (0.7)	7.4 (0.8)	0.017
PSV Level (cmH_2_O), median [Q1,Q3]	5.0 [5.0,5.0]	5.0 [5.0,7.5]	<0.001	5 [5.0, 5.0]	5 [5.0, 5.0]	1.000
MV durations (h), median [Q1,Q3]	15.9 [7.2,37.0]	36.9 [15.0,89.6]	<0.001	16.0 [13.0,20.0]	36.0 [16.8,61.0]	<0.001
SBT success times, *n* (%)						
0	7,803 (58)	1,677 (61)	<0.001	0 (0.00)	0 (0.00)	<0.001
1	3,645 (27)	531 (19)		449 (100)	45 (88)	
2	1,025 (8)	230 (8)		2 (0)	4 (8)	
≥3	960 (7)	318 (12)		0 (0.00)	2 (4)	
Urine output (mL/kg/h), median [Q1,Q3]	0.9 [0.6,1.5]	0.7 [0.3,1.2]	<0.001	1.5 [1.2,1.9]	1.4 [1.1,1.6]	0.024
Antibiotic types, *n* (%)						
0	10,288 (77)	1,764 (64)	<0.001	0 (0.00)	0 (0.00)	1.000
1	2,192 (16)	428 (16)		451 (100)	51 (100)	
2	752 (6)	334 (12)		0 (0.00)	0 (0.00)	
3	169 (1)	169 (6)		0 (0.00)	0 (0.00)	
≥4	32 (0)	61 (2)		0 (0.00)	0 (0.00)	
Failure type, *n* (%)
Death	/	1,504 (55)		/	4 (8)	
NIV	/	411 (15)		/	43 (84)	
Reintubation	/	902 (33)		/	14 (27)	

*Values are presented as mean (SD) if not otherwise specified. MIMIC-IV, Medical Information Mart for Intensive Care-IV; ZS, Zhongshan; BMI, body mass index; MAP, mean arterial pressure; SpO_2_, peripheral oxygen saturation; CVP, central venous pressure; PEEP, positive end-expiratory pressure; PSV, pressure support ventilation; MV, mechanical ventilation; SBT, spontaneous breathing trial; NIV, non-invasive ventilation*.

### Development of CatBoost Model

**Figure 3:**
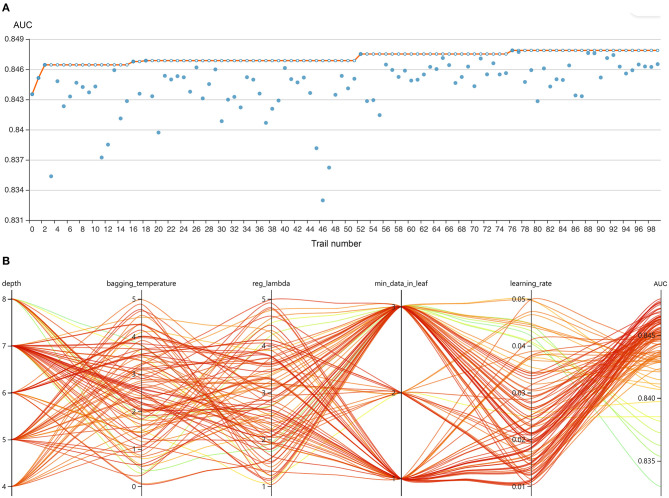
Hyperparameter optimization using an automated machine learning toolkit. **(A)** Each blue point represents the result of a trial, and the dark orange line represents the best area under the receiver operating characteristic curve (AUROC). **(B)** Each line represents a trial, and the green to red color represents its AUROC.

The RFE algorithm was performed, and 19 key features were finally selected, including age, body mass index (BMI), stroke, heart rate, respiratory rate, mean arterial pressure (MAP), SpO_2_, temperature, pH, central venous pressure (CVP), tidal volume, positive end-expiratory pressure (PEEP), mean airway pressure, PSV level, MV duration, spontaneous breathing trial (SBT) success time, urine output, crystalloid amount, and antibiotic types. Hyperparameter optimization was then conducted (shown in Figure 3). After 100 trials, a CatBoost model with the greatest AUROC was obtained. The final settings of the hyperparameter search are listed in Supplementary Table 2.

**Figure 4:**
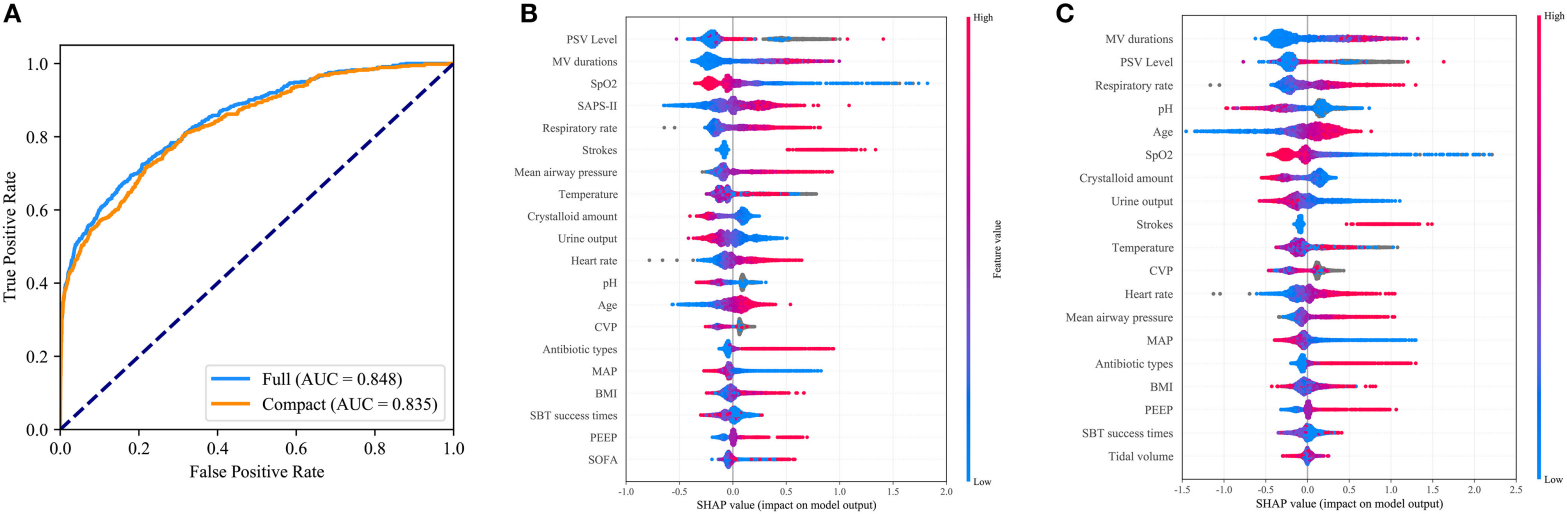
Comparison of the full and the compact CatBoost models. The full model was developed based on all available features while the compact was derived based on key features selected by the recursive feature elimination algorithm. Both models had optimized hyperparameters. **(A)** Receiver operating characteristic curves (ROCs) of the full and the compact models. Distribution of the impacts each feature had on the output of the full model **(B)** or compact model **(C)** estimated using the SHapley Additive exPlanations (SHAP) values. The plot sorts features by the sum of SHAP value magnitude over all samples. The blue to red color represents the feature value (red high, blue low). The x-axis measures the impacts on the model output (right positive, left negative).

As shown in Figure 4A, the CatBoost model with all available variables had a remarkable AUROC of 0.848, while the compact model with 19 selected variables had a slightly lower AUROC of 0.835. SHAP values for the two models were assessed in the internal validation set, and are shown in Figures 4B,C, respectively. Feature values were indicated by a spectrum with blue representing the lowest value. A positive SHAP value represents an increase in the risk of EF and vice versa. Features were ranked according to the sum of absolute SHAP values over all samples. As shown, MV duration is the most important feature for prediction of EF in the final model, and a longer duration indicates a higher EF risk.

**Figure 5:**
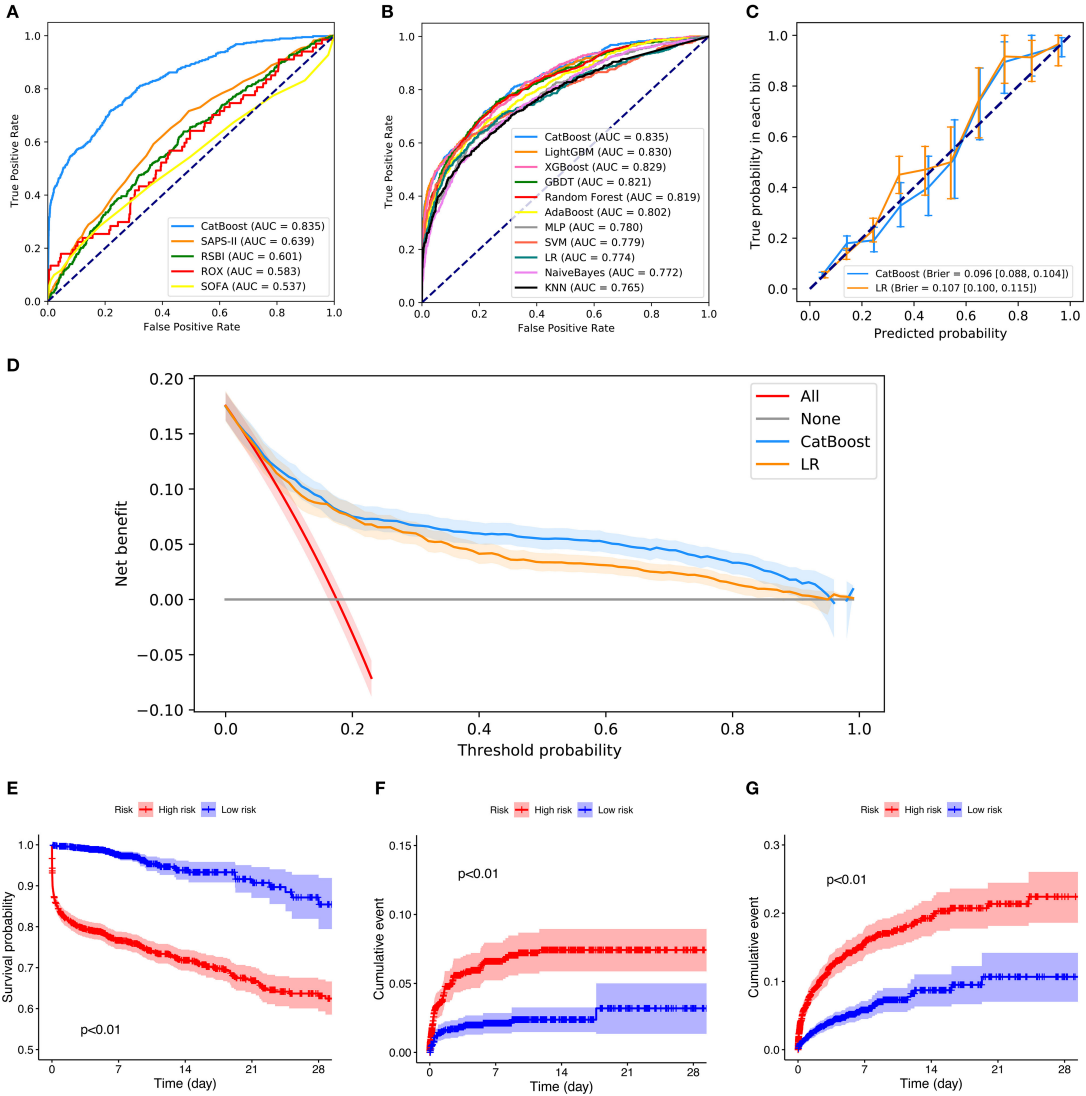
Comparison of model performance in the internal validation set. **(A)** Receiver operating characteristic curves (ROCs) of CatBoost and other predictive factors. **(B)** Receiver operating characteristic curves (ROCs) of different models. **(C)** Calibration plot of CatBoost model and Logistic Regression (LR). **(D)** Decision curve analysis of CatBoost and LR. **(E)** Kaplan Meier analysis of 28-day survival in the high- and low-risk groups; **(F)** Cumulative NIV events in the high- and low-risk groups; **(G)** Cumulative reintubation events in the high- and low-risk groups. AUC, area under the curve; RSBI, Rapid Shallow Breathing Index; SOFA, Sequential Organ Failure Assessment; SAPS-II, Simplified Acute Physiology Score-II; ROX, the ratio of pulse oximetry/fraction of inspired oxygen to respiratory rate; XGBOOST, eXtremely Gradient Boosting; GBDT, Gradient Boosting Decision Tree; KNN, K-Nearest Neighbor; SVM, Support Vector Machine; MLP, Multi-Layer Perceptron; LR, Logistic Regression; PPV, Positive Predictive Value; NPV, Negative Predictive Value.

Figures 5A,B depicts the comparison between the CatBoost model and other predictive factors or models. As shown, our CatBoost model significantly outperformed other predictive factors or models and had the greatest AUROC. To further elucidate the performance of our model, a calibration plot (Figure 5C) and decision curve analysis (Figure 5D) were performed (29). For simplicity, only the results of CatBoost and LR are demonstrated. The sensitivity and specificity analysis of these predictive methods in the internal validation set is summarized in Table 2. Although the CatBoost model was not the best on all measures, it had the greatest Youden Index (0.499) which is considered a more comprehensive evaluation approach.

**Table 2:** Model performance in the internal and prospective validation sets.

**Model**	**AUROC**	**Best cutoff**	**Gray zone**	**Values in gray zone**	**Youden index (%)**	**Sensitivity (%)**	**Specificity (%)**	**PPV (%)**	**NPV (%)**
**Internal validation**									
CatBoost	**0.84 (0.82–0.85)**	0.148	0.07**–**0.24	1,276 (39.60%)	**50**	72 (68**–**76)	78 (76**–**79)	41 (38**–**44)	**93 (92–94)**
LightGBM	0.83 (0.81**–**0.85)	0.147	0.06**–**0.24	1,269 (39.39%)	49	70 (66**–**74)	79 (77**–**80)	41 (38**–**44)	93 (92**–**94)
XGBoost	0.83 (0.81**–**0.85)	0.156	0.04–0.23	1182 (36.69%)	47	64 (60–68)	84 (82–85)	45 (42–49)	92 (91–93)
GBDT	0.82 (0.80–0.84)	0.144	0.08–0.25	1380 (42.62%)	50	**76 (72–79)**	74 (73–76)	38 (36–41)	93 (92–95)
Random forest	0.82 (0.80–0.84)	0.183	0.08–0.29	1472 (45.46%)	49	73 (70–77)	75 (74–77)	39 (36–42)	93 (92–94)
AdaBoost	0.80 (0.78–0.82)	0.493	0.49–0.50	1046 (32.30%)	45	61 (57–65)	84 (83–86)	45 (41–49)	91 (90–92)
MLP	0.78 (0.76–0.80)	0.173	0.02–0.35	1737 (53.64%)	43	63 (59–67)	80 (79–82)	40 (37–43)	91 (90–92)
SVM	0.78 (0.76–0.80)	0.142	0.09–0.16	2004 (61.89%)	46	60 (56–64)	**86 (85–87)**	**47 (44–51)**	91 (90–92)
LR	0.77 (0.75–0.80)	0.179	0.06–0.25	1840 (56.83%)	44	64 (60–68)	80 (79–81)	40 (37–43)	91 (90–92)
NaiveBayes	0.77 (0.75–0.79)	0.058	0.00–0.49	2711 (83.72%)	41	65 (62–70)	75 (74–77)	36 (33–39)	91 (90–92)
KNN	0.77 (0.74–0.79)	0.188	0.05–0.21	1428 (44.10%)	40	55 (51–59)	85 (84–86)	44 (40–47)	90 (89–91)
**Prospective validation**									
CatBoost	**0.80 (0.74–0.86)**	0.049	0.04–0.09	198 (39.36%)	**48**	85 (74–93)	64 (59–68)	21 (15–26)	97 (95–99)
LR	0.77 (0.70–0.84)	0.834	0.37–0.88	246 (48.91%)	38	51 (37–65)	87 (84–90)	31 (21–42)	94 (92–96)
LightGBM	0.77 (0.70–0.84)	0.053	0.04–0.10	260 (51.69%)	44	81 (69–91)	63 (59–68)	20 (15–26)	97 (95–99)
XGBoost	0.77 (0.71–0.82)	0.045	0.03–0.13	217 (43.14%)	48	83 (71–93)	65 (61–70)	21 (15–27)	97 (95–99)
SVM	0.74 (0.67–0.82)	0.956	0.33–0.85	254 (50.50%)	38	41 (28–55)	97 (95–98)	60 (43–77)	94 (91–96)
NaiveBayes	0.74 (0.66–0.80)	0.377	0.42–0.87	230 (45.73%)	35	**96 (90–100)**	39 (34–43)	15 (12–19)	**99 (97–100)**
GBDT	0.72 (0.64–0.79)	0.495	0.34–0.85	261 (51.89%)	30	81 (68–91)	49 (44–54)	15 (11–19)	96 (93–98)
MLP	0.71 (0.64–0.78)	0.781	0.37–0.90	275 (54.67%)	31	55 (42–69)	76 (72–80)	20 (14–27)	94 (91–96)
KNN	0.71 (0.65–0.78)	0.63	0.42–0.88	239 (47.51%)	33	69 (55–81)	65 (60–69)	18 (13–24)	95 (92–97)
AdaBoost	0.70 (0.62–0.78)	0.992	0.34–0.88	271 (53.88%)	30	31 (19–44)	**98 (97–100)**	**70 (50–88)**	93 (90–95)
Random forest	0.69 (0.62–0.77)	0.64	0.32–0.85	278 (55.27%)	33	48 (31–58)	85 (74–92)	60 (49–72)	93 (91–95)

*Models are ordered according to their areas under receiver operating characteristic curves. Youden index was defined as sensitivity + specificity – 1. The bold values indicate the best performance of the 10 models in the internal or prospective validation. XGBOOST, eXtremely Gradient Boosting; GBDT, Gradient Boosting Decision Tree; KNN, K-Nearest Neighbor; SVM, Support Vector Machine; MLP, Multi-Layer Perceptron; LR, Logistic Regression; PPV, Positive Predictive Value; NPV, Negative Predictive Value*.

Additionally, patients in the internal validation set were divided into high- and low-risk groups, according to whether their failure risks predicted by CatBoost were greater than the median risk in the set. Figures 5E–G shows the survival curves, cumulative NIV curves, and cumulative reintubation curves of the two groups, respectively. Log rank *p*-values are lower than 0.01 in Figures 5E–G, indicating significant differences between the high- and low-risk groups.

### Prospective Validation and a Web-Based Tool

**Figure 6:**
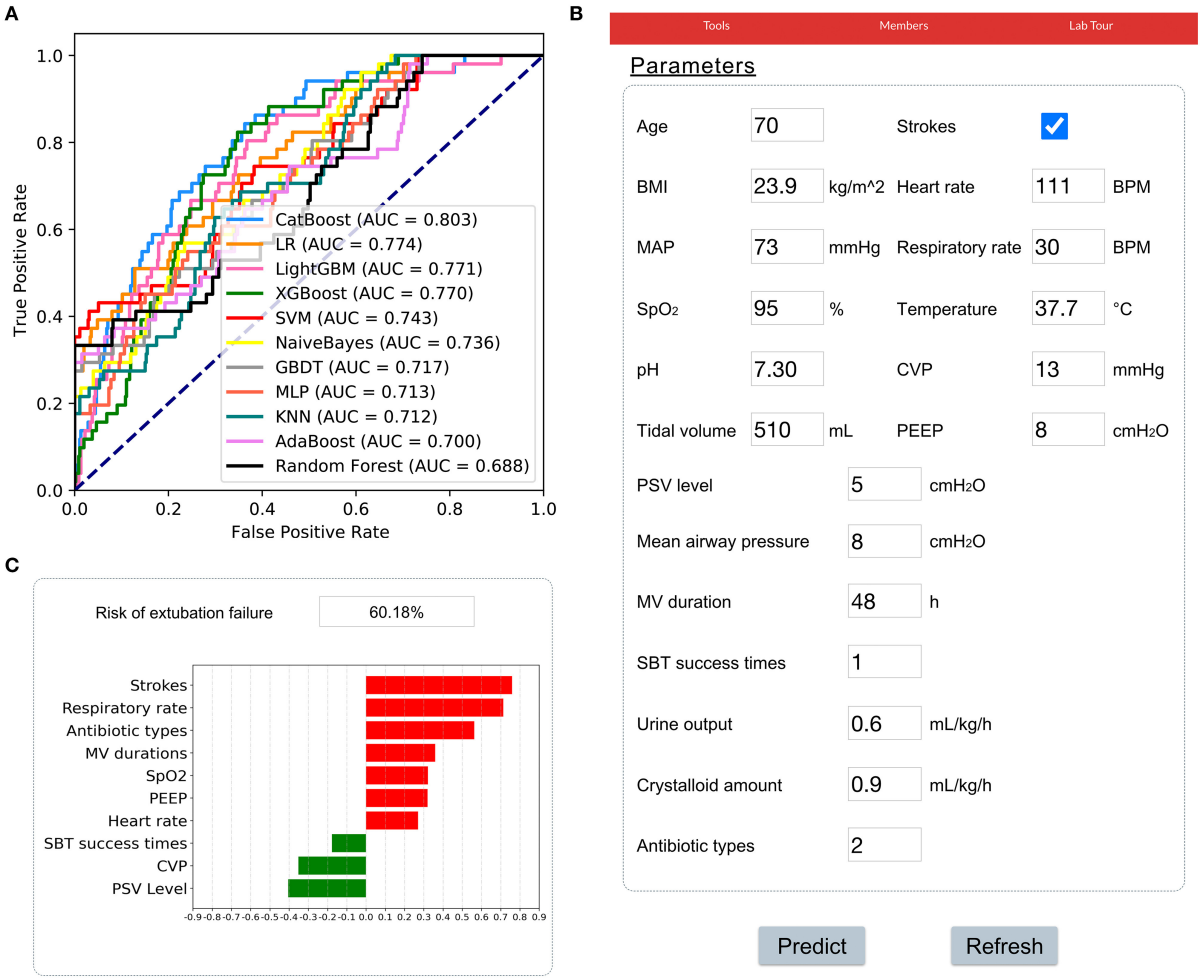
Application of the CatBoost model. **(A)** Receiver operating characteristic curves of different models in the prospective validation set. **(B)** An example of the web-based tool. **(C)** The prediction results of the CatBoost model and the top 10 important features were summarized. A green bar indicates a protective factor while a red bar represents a risk factor. The bar length corresponds to the magnitude of protection or risk.

The results of prospective validation are shown in Figure 6A. It can be seen that our model also had a better generalization ability (AUROC: 0.803 [95%CI: 0.74–0.86]) than the other models. The sensitivity and specificity analyses are summarized in Table 2.

In addition, a web-based tool was established for clinicians to use the compact model, http://www.aimedicallab.com/tool/aiml-extfailure.html. An example of using our tool is depicted in Figure 6B. A user needs to enter the variable values when weaning, leaving missing values blank and clicking the “predict” button. The risk of EF assessed by the CatBoost model, and the top 10 important features will be shown to the user, as shown in Figure 6C.

## Discussion

In this study, we developed and validated an accurate machine-learning model for predicting EF in ventilated critically ill patients. To our knowledge, this is the first model constructed on a large-scale public database and then further validated in a university teaching hospital prospectively. Moreover, different to previously published models, we provide an open and accessible data interface for the public to use and validate our model.

Eighty-nine variables were evaluated, and key features were screened out, improving model usability compared with previous studies. We eventually selected 19 key features that could be more easily obtained, including age, BMI, stroke, heart rate, respiratory rate, MAP, SpO_2_, temperature, pH, CVP, tidal volume, PEEP, mean airway pressure, PSV level, MV duration, SBT success time, urine output, crystalloid amount, and antibiotic types. As expected, the slight decrease in the AUROC of the compact model based on selected features (shown in Figure 4A), demonstrated that other variables could be excluded without a marked negative effect on the model performance.

Previous studies indicated that age and BMI are two important factors associated with an increased risk of EF (6, 30–32). Elderly or overweight patients have a higher prevalence of comorbidities, a decline in cardiac and lung functions, and a higher risk of respiratory failure, leading to a worse outcome following extubation. Increasing evidence supports that stroke patients suffer a higher risk of EF, and airway management remains a clinical challenge in this population (33, 34).

In addition, abnormal vital signs, such as heart rate, respiratory rate, MAP, SpO_2_, and temperature were related to a higher EF risk (35, 36). These basic factors are commonly used in ICUs, representing the vital status of a patient, and were included in many prediction models. Arterial pH was another key feature in our study, which monitors the body's acid-base balance. A lower-than-normal pH indicates hypoventilation or severe pulmonary disease, and was a remarkable predictive factor for EF according to its SHAP values.

Our study also showed that CVP contributed to EF prediction. As shown in Figure 4C, gray points of CVP representing missing values, had positive SHAP values as shown, which suggested that patients without CVP measures had a higher failure rate. Prior research has explored the benefit of CVP measurement in septic patients (37). In our study, it was shown that CVP monitoring might also be associated with improved outcomes following extubation. More studies are needed to confirm this.

As expected, SBT success time and parameters of MV such as tidal volume, PEEP and mean airway pressure, helped to accurately predict EF in our study. By assessing SHAP values, we found that MV duration and PSV level were the most important features for prediction, which is consistent with previous studies (7, 38–41). Additionally, fluid balance (only urine output, crystalloid amount in our study) and antibiotic types were included in the final model. Evidence suggests that fluid balance was associated with failed extubation and was consistent with our findings (32, 42). The number of antibiotics administered to a patient reflected his or her infectious status. As shown in Figure 4C, a greater number of antibiotics administered was related to a higher EF risk.

Although SAPS-II, APACHE-II, and other risk scores showed great importance for prediction in previous studies (16, 17) as well as in our study, we excluded these features for two main reasons. Firstly, the extracted features covered most components of these scores, leading to negligible benefits of including these scores. Previous research has shown that excluding these scores did not impede the development of an accurate model (43). Secondly, including these scores such as APACHE-II and SOFA, would make our model inconvenient to use in clinical settings.

Based on these key features, a CatBoost model was derived with optimized hyperparameters and outperformed other predictive factors and 10 models in the MIMIC-IV dataset. CatBoost, a member of the gradient boosting algorithm family, has not been widely adopted in critical care research, despite the fact that CatBoost significantly outperformed other machine-learning models in various tasks in some previous studies (44). Its main advantage is that it can successfully handle categorical features and missing values automatically, and takes advantage of dealing with them during training instead of preprocessing time (45). Therefore, categorical features no longer need to be encoded, and missing values do not need to be imputed. Another advantage of the algorithm is that it uses a new schema to calculate leaf values when selecting the tree structure. The schema helps to reduce overfitting, the major problem that constrains the generalization ability of machine-learning models (45).

Apart from internal validation, we enrolled more than 500 patients in the CSICU of Zhongshan Hospital, Fudan University to prospectively validate our model. As shown in Figure 6, our model had a greater AUROC than others, indicating a remarkable generalization ability and clinical value. To help clinicians use the model, a web-based tool was developed, which provides a user-friendly interface. After entering the variables, the risk of EF, as well as the top 10 important features were shown. These results will help clinical decision-makers to understand the patient's status and prepare an appropriate treatment strategy.

More importantly, our model is a promising tool for improving the prognosis of patients who undergo extubation and can have a positive impact both medically and financially. As shown in previous studies, either EF or reintubation is independently associated with higher mortality (3, 46). Reintubation is also accompanied by the occurrence of complications such as acute respiratory distress syndrome, sepsis, ventilator-associated pneumonia, prolonged ICU stay, and increased medical cost (4, 5). By adopting this model, if a patient is predicted to have a high risk of EF, weaning from MV can be delayed, and more intensive monitoring will be granted, which may avoid injuries caused by EF and reduce mortality. In addition, extra medical costs due to further medical investigations and treatments could be prevented as low-risk patients would be less likely to develop severe complications. The clinical value of this model will be further assessed and reported in future prospective studies.

Several limitations of this study should be considered. Firstly, there is still disagreement on the definition of EF. The definition adopted in the present study included the need for NIV, reintubation and death within 48 h following extubation. High-flow oxygen therapy, with the potential to prevent reintubation, was excluded. Further studies should be carried to include the use of a high-flow nasal cannula as EF. A different time interval (e.g., 72 h following extubation) could also be studied. Secondly, the majority of routine ventilation methods following surgery were included in our study, which have a minimal risk of EF. This could have led to biased results. Our future study is to fine-tune our model or develop new models for patients who undergo difficult or prolonged weaning. These patients have a significantly higher risk of EF in ICUs. Thirdly, novel parameters or techniques proposed in recent studies were not included in the present study, such as central venous-to-arterial P_CO2_ difference (36), the cuff leak test (47), thenar oxygen saturation (48), and diaphragm dysfunction (49). We argue that these parameters or techniques need multiple measurements or complex calculations, leading to difficult application in clinical settings. The variables selected in our study are rapidly available and directly measured, improving model practicality. Fourthly, the sensitivity and specificity of our model were 72 and 78%, respectively, indicating that the false negative rate could be relatively high. A number of patients with EF may be missed, which is important as they have a non-negligible mortality. Lastly, patients enrolled in the prospective validation set were all from one CSICU; thus, this dataset can only validate the efficacy of our model in a limited patient population. More large-scale prospective studies are needed to validate our model.

## Conclusions

In conclusion, the present study screened out 19 key features associated with EF and developed a CatBoost model which can better predict EF than other predictive methods in ICUs.

## Data Availability Statement

The MIMIC-IV data were available on the project website at https://mimic-iv.mit.edu/. But the validation set generated for this article is not readily available because the ethics committee does not allow the release of the data. Requests to access the dataset should be directed to Guo-Wei Tu, tu.guowei@zs-hospital.sh.cn.

## Ethics Statement

The establishment of the MIMIC-IV database was approved by the Massachusetts Institute of Technology (Cambridge, MA) and Beth Israel Deaconess Medical Center (Boston, MA), and consent was obtained for the original data collection. Therefore, the ethical approval statement and the need for informed consent were waived for the studies on this database. Besides, the prospective study involving human participants was reviewed and approved by Ethics Committee of Zhongshan Hospital, Fudan University. The patients/participants provided their written informed consent to participate in this study.

## Author Contributions

Q-YZ, HW, and J-CL: conception, design, data analysis, and interpretation. G-WT and ZL: administrative support. Q-YZ and HW: collection and collation of data. All authors: manuscript writing and final approval of manuscript.

## Conflict of Interest

The authors declare that the research was conducted in the absence of any commercial or financial relationships that could be construed as a potential conflict of interest.

## Supplementary Material

Supplementary Table 1 Predictors extracted in MIMIC-IV.

Supplementary Table 2 Hyperparameter search domains and final settings.
