# Supplementary material for: Development and Validation of a Machine-Learning Model for Prediction of Extubation Failure in Intensive Care Units
Source: Front Med (Lausanne). 2021 May 17;8:676343. doi: 10.3389/fmed.2021.676343 (PMC8165178; doi:10.3389/fmed.2021.676343)
Supplement: Supplementary Table 2 — Hyperparameter search domains and final settings. [file Table_2.DOCX]

Table S2. Hyperparameter search domains and final settings

| Hyperparameters | Type | Search domain | Final setting |
| --- | --- | --- | --- |
| 'depth' | Choice | {4, 5, 6, 7, 8} | 7 |
| 'bagging_temperature' | Uniform | [0, 5] | 2.53 |
| 'reg_lambda' | Uniform | [1, 5] | 2.17 |
| 'learning_rate' | Uniform | [0.01, 0.05] | 0.0196 |
| 'min_data_in_leaf' | Choice | {1, 2, 3} | 3 |
